# Reducing protected lands in a hotspot of bee biodiversity: bees of Grand Staircase-Escalante National Monument

**DOI:** 10.7717/peerj.6057

**Published:** 2018-12-04

**Authors:** Joseph S. Wilson, Matt Kelly, Olivia Messinger Carril

**Affiliations:** 1 Department of Biology, Utah State University—Tooele, Tooele, UT, USA; 2 Branchport, NY, USA; 3 Santa Fe, NM, USA

**Keywords:** Bee conseration, Biodiversity, National monuments, Public lands, Native bees, Pollinators

## Abstract

Grand Staircase-Escalante National Monument is a federally protected area found in central southern Utah. Designated in 1996 by President William J. Clinton, it was recently reduced in size by President Donald J. Trump in a proclamation that turned the one large monument into three smaller ones. A long-term, standardized study of the bees had been conducted from 2000–2003, revealing 660 species. The bee communities of the area are characterized by being spatially heterogeneous; most of the bees occur in isolated areas, with only a few being both abundant and widespread. Here we examine what affect the recent resizing of the monument has on the number, and ecology, of the bees now excluded from monument boundaries. Using the new monument boundaries and the geographic coordinates associated with each bee, we derived new species lists for each of the three monuments, and compared them to each other, and to the excluded lands. All three monuments now protect unique faunas, with Bray–Curtis similarity values not exceeding 0.59%. Each monument now harbors species not found in the other two monuments. We found that 84 bee species are no longer protected by any of the three monuments. These 84 species were not concentrated in one area that is now excluded, but were scattered throughout the newly excluded lands. For some of the excluded bee species, there is no evidence that they are rare or imperiled, being widespread throughout the west. However, there is a concentration of bees in the southern and eastern former monument lands that represent range extensions from nearby hot deserts. In addition to numerous range extensions, the list of excluded bees also contains several undescribed species (newly discovered species that have not yet been named and described by taxonomists) and morphospecies (individuals that are morphologically distinct, but that require additional research before species designations can be made). This indicates that the bee communities housed in these excluded areas would benefit from additional scientific inquiry. The areas now excluded from monument protections house a greater proportion of the original GSENM bee community than any of the three new monument units. We conclude this paper by discussing what the smaller monuments might mean for bee conservation in this hot spot of bee biodiversity and suggest that bee communities here and elsewhere should be taken into account when conservation decisions are being made.

## Introduction

Protected federal lands, such as national monuments, national parks, and wilderness areas are important refugia for biodiversity (Lorah & Southwick, 2003; Strittholt & Dellasala, 2001), are less susceptible to habitat modification than other lands (Andam et al., 2008; Hoekstra et al., 2005), and help to maintain ecosystem services that extend beyond the edges of the protected lands (Dudley et al., 2011; Hansen & DeFries, 2007). The establishment of these protected lands, however, is not without controversy (Jakus & Akhundjanov, 2018). Arguments against them include the ideas that federal lands represent government overreach (Popper, 1988; Rusnak, 2003), limit the ability to extract natural resources (Foley, 1997; Sellars, 2009), and are unable to protect the organisms and landscapes within (Cushman, Landguth & Flather, 2012; Havlick, 2002).

Among the first monuments to be established through the federal Antiquities Act of 1906 was Grand Canyon National Monument by President Theodore Roosevelt in 1908 (Wolf & Mast, 1998). Not long after the establishment of that monument, the federal government was sued in order to allow mining in the newly protected lands. The Supreme Court unanimously ruled in Cameron v. United States (1920) that Grand Canyon National Monument was “an object of scientific interest” and was thereby a valid reassigning of public lands, and exempt from mining. Since the passing of the Antiquities Act, 117 national monuments have been established.

In 1996, President William J. Clinton designated 1.9 million acres in southern Utah as Grand Staircase-Escalante National Monument (GSENM) (Clinton, 1996). The justification for creating this monument was in part a recognition that this area houses unique geological, paleontological, cultural, and biological resources worth preserving—including numerous endemic plants and their pollinators (Clinton, 1996).

Grand Staircase-Escalante National Monument spans three unique physiographic regions (Grand Staircase, Kaiparowits Plateau, and The Canyons of the Escalante), and includes habitats ranging from alpine coniferous forests to desert shrublands. Research to date indicates that GSENM is a biologically diverse landscape (Flinders et al., 2002; Oliver, 2003), supporting a rich and distinct flora (Bashkin et al., 2003; Shultz, 1998). GSENM also houses a diverse wild bee fauna (Carril et al., 2018), with over 18% of the total bee fauna for the United States occurring in its boundaries, including 49 undescribed bee species (newly discovered species that have not yet been described and named by taxonomists) (Carril et al., 2018).

In 2017, President Donald J. Trump issued a proclamation that reduced the size of GSENM by nearly 50% and divided the monument into three distinct management units (Trump, 2017), with roughly one unit in each of the three physiographic regions. The proclamation claims that most of the habitat for animal and plant species specifically mentioned as objects of scientific or historic interest in the 1996 proclamation are still contained in the new boundaries, and that areas now outside of monument boundaries were “unnecessary for the care and management of the objects to be protected within…” Despite their ecological importance and high diversity in the region, and despite their attention in other presidential proclamations (Obama, 2014), pollinators, including native bees, were not mentioned in this most recent proclamation.

The diminishment of protected land raises important questions about the efficacy of a smaller area in protecting the same organisms that a larger area was set aside to protect (Wilcox & Murphy, 1985). With bees in particular, this is important for two reasons. First, the bee communities studied in GSENM are spatially heterogeneous, with most species occurring at only a few localities within the original monument boundaries. Second, one of the unique features of the original monument boundaries was that it included, at its southern extent, Mojave Desert floral elements and a suite of uncommon bees associated with this desert; along its northern and eastern boundaries were found rare bees more often associated with montane habitats.

Based on these observations, we investigate here how the modifications of the GSENM boundaries will affect this diverse bee fauna. Specifically: (1) What percentage of the original bee fauna is still included within protected areas? (2) How have the new designations affected alpha- and beta-diversity for the monument? (3) What bees are no longer within protected federal lands, and what do we know about them?

We focus primarily on the faunal differences created by the new boundaries with the presumption that species still found within the new monument units are more likely to be protected than those outside of the new units. As an appendix, we include bee species lists for each of the three new units, to replace the species list recently published for the monument as a whole (Table S1, Carril et al., 2018).

## Materials & Methods

**Figure 1:**
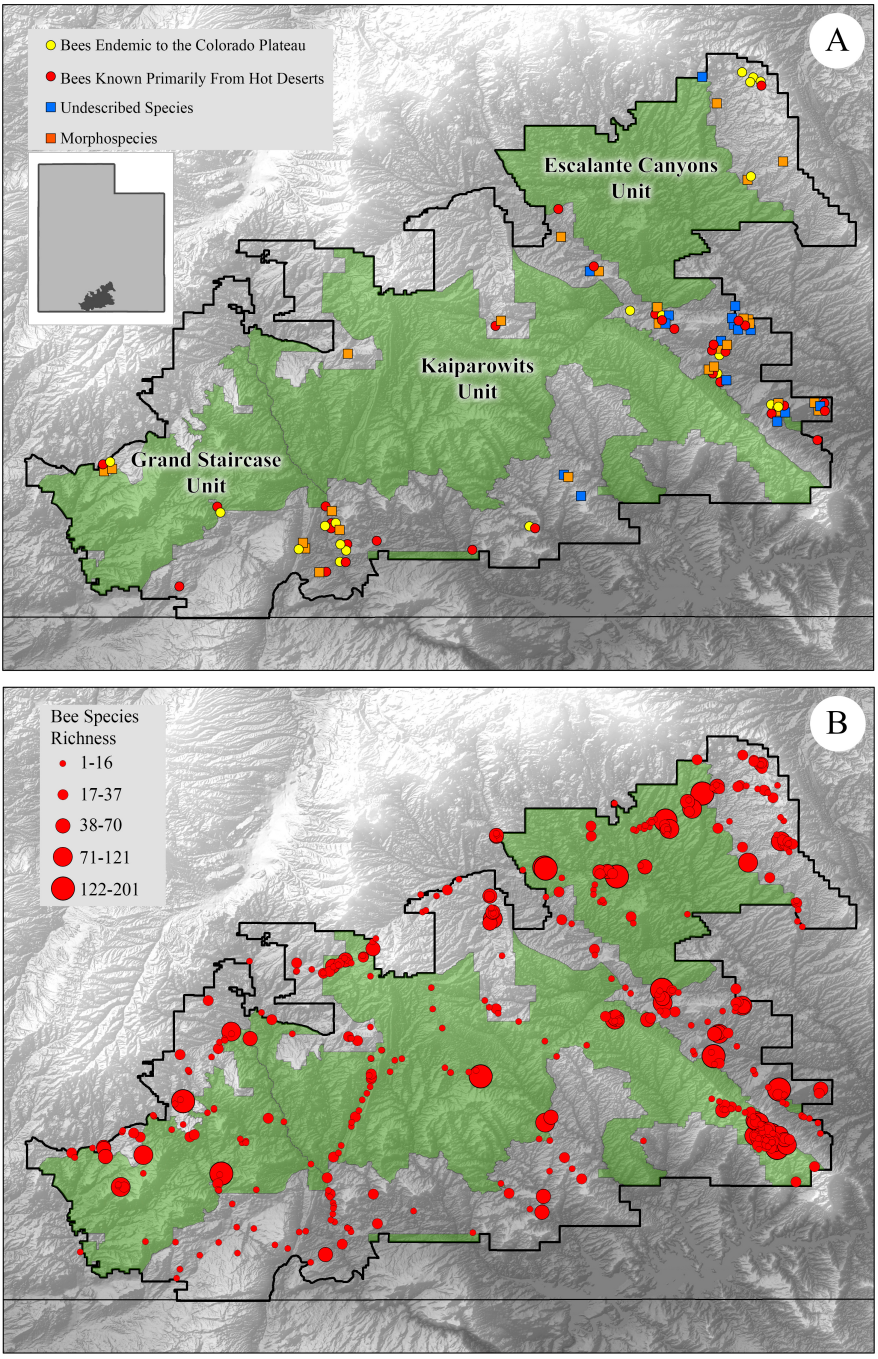
Map of GSENM showing original boundaries as well as the new reduced boundaries with bee collection sites marked. (A) Map of GSENM showing original boundaries as well as the new reduced boundaries (in green). Select bee groups no longer found in monument boundaries are marked. Bee species endemic to the Colorado Plateau marked with yellow circles, bee species known primarily from hot deserts (Mojave, Sonoran, Chihuahuan) are marked with red circles. Undescribed species are marked with blue squares and morphospecies are marked with orange squares. (B) Collection localities from Carril et al. (2018) shown with circles representing bee species richness known from each site, larger circles represent sites that house richer bee communities.

In total, 660 bee species, in 55 genera, and six bee families, are known from the original GSENM (Carril et al., 2018). We compared bee communities originally reported from GSENM to those in each new management unit (Grand Staircase, Kaiparowits, Escalante Canyons). We also examined the list of bees excluded from the monument (Fig. 1A). Bees were mapped onto old and new boundaries using ArcGIS 9.2 (ESRI, Inc., Redlands, CA, USA). Faunal species lists, with abundance, were compiled of bees in the original boundaries and in the new boundaries to determine how bee communities in the newly structured areas compared to the bee fauna from the original monument.

Ecological traits of individual bee species were assessed based on collection data (Carril et al., 2018) and from other large datasets (i.e., https://www.discoverlife.org/: Ascher & Pickering, 2018; Krombein, 1979). If a bee was abundant in multiple localities and was only collected from one plant genus or plant family, we considered it a potential specialist. If a bee species was collected on numerous different plant families, we considered it a generalist. Many bees were only represented by a few individuals; no host preferences were determined for these species because not enough data were available. While floral specialization is best determined by palynological examinations, these have not been done for bees of GSENM. To determine the distributions of excluded bee species, each species was searched on https://www.discoverlife.org/, which pulls data from a number of large insect collections located around North America, and range maps were examined. For example, if a bee was primarily found in the North American hot deserts (Mojave, Sonoran, or Chihuahuan) it was considered a hot desert endemic and its presence in GSENM represented a range extension.

Similarity values between monument units were calculated using a Bray–Curtis similarity index (Bray & Curtis, 1957). To account for the unevenness in collecting between the three units (*N* = 26,359 Escalante Canyons unit; *N* = 7,812 Grand Staircase unit; *N* = 28,461 Kaiparowits unit; *N* = 19,092 Excluded lands), a bootstrapping approach was used. Specifically, 7,812 individuals were randomly selected from each area and used to populate the lists used to calculate Bray–Curtis similarity values between each unit. This process was repeated 1,000 times to obtain an average similarity among sites.

## Results

Of the 660 total species known from the original GSENM, 576 (87.3%) of them are found in at least one of the three new units (Table S1). On the other hand, 84 species (12.7%) originally found in GSENM are no longer found in monument lands. The Kaiparowits unit houses the richest bee fauna, with 449 species (68.0% of the original GSENM fauna). Escalante Canyons houses 393 bee species (59.4% of the original). Grand Staircase contains 313 species (47.4% of the original). The lands now excluded from any of the new monuments house 521 bee species, representing 78.9% of the original GSENM bee fauna.

Each of the new units harbors a relatively distinct bee community as indicated by the similarity values between monuments (Table 1). In addition to having the richest bee fauna, Kaiparowits also has the most distinct fauna, and was less similar to either of the other two units than they were to each other. The highest similarity between two units (Escalante Canyons and Grand Staircase, on either side of Kaiparowits), however was just 0.583 (with 1 being completely similar and 0 being not similar at all).

**Table 1:** Similarity values comparing bee communities. Average Bray–Curtis similarity values between each newly designated protected unit based on a bootstrapping analysis of *N* = 7,812 individuals, with 1,000 replicates. Values approaching 1 indicate highly similar bee communities.

	**Escalante Canyons**	**Kaiparowitz**	**Grand Staircase**
**Escalante Canyons**	–	0.472	0.583
**Kaiparowitz**		–	0.582

The bees excluded from monument lands share few biological characteristics in common. For example, of the 84 excluded bee species, 30 of them are generalists (collecting pollen from a wide variety of unrelated plant taxa) and 30 appear to be floral specialists (foraging only from a limited number of plant taxa, usually in the same plant genus or family) based on collection data. None of the specialists visit plants that are endemic to GSENM. However, there are 24 species that are poorly studied and have unknown floral preferences (Table S2).

Many of the species no longer found in monument boundaries represent notable range extensions based on known distributions (Ascher & Pickering, 2018). Seventeen of the excluded species are primarily found in North American hot deserts (Mojave, Sonoran, or Chihuahuan) and the records from GSENM represent their northernmost populations. Six other excluded species are endemic to the Colorado Plateau, the ecoregion that encompasses GSENM.

No single area of the former GSENM contains all of the species now excluded from monument lands. Rather, the excluded bees occur widely in areas outside the new boundaries. However, there are a few areas that are no longer included in the new monument units that have high concentrations of endemic, rare, undescribed, or morphospecies (Fig. 1A). Morphospecies are individuals that are morphologically distinct and require additional research before species designations can be made—some of these occur only in the monument, and others are widespread and known from collections across the US; they exist in part because taxonomic revisions have not been completed for all bee groups. In the northeastern corner of the monument, near higher elevation mountainous regions, we found a concentration of morphospecies and Colorado Plateau endemics. Along the ‘Hole-in-the-Rock’ corridor, which separates the new Escalante Canyons unit from the Kaiparowits unit, are hot desert endemics, as well as a majority of the undescribed species, and 14 of the 25 morphospecies (Fig. 1A). Finally, a region near the southwestern edge of the former GSENM contains 8 of the 25 morphospecies, and houses many hot desert endemics who reach the northern extent of their range here (Fig. 1A).

Over 40% of the bees now excluded from monument boundaries are rare, with only a single specimen collected during the four years of the study (Table S2). At a broader geographic level, a few of the bees that were rare in the original GSENM boundaries are also rare across the western United States. In contrast, other bees that were rare in GSENM are widespread in other areas of the west.

Many of the collecting sites known to house diverse bee faunas (Carril et al., 2018) are still within the new monument boundaries, but 10 of the richest and most diverse sites found in GSENM have been excluded from monument lands, many of these are along the ‘Hole-in-the-Rock’ corridor (Fig. 1B).

## Discussion

Our analysis of the new national monument units shows that they encompass much of the bee richness formerly protected by the old monument boundaries, with the Kaiparowits Plateau unit harboring the greatest diversity. Still included within the boundaries are bees that are endemic to the Colorado Plateau, as well as new state records, newly discovered species, and other bees that represent significant range extensions (Table S1, and Carril et al., 2018). Our data show that the original GSENM housed a richer bee community than the new smaller monument units. A long-standing issue among conservation biologists delves into whether a single large protected area or several small ones in the same area is better suited to protecting biodiversity (the SLOSS debate, Diamond, 1976). Each situation likely warrants a different approach and must consider many factors including the degree to which included or excluded species are imperiled, the likelihood of genetic isolation, the mobility of the organisms, their resource requirements, the degree of disturbance in the unprotected lands, and the proximity of the smaller reserves to each other as well as their size. Bees are central place foragers with small home ranges and often limited dispersal ability (Greenleaf et al., 2007). They are also haplodiploid, and the likelihood of genetic isolation is increased as a result (Zayed, 2009; Davis et al., 2010). And in at least some species, lower genetic diversity can lead to decreased resilience to pathogens (Whitehorn et al., 2011). Finally, in GSENM we have typically found bees to be locally abundant, but not widespread. These factors suggest that, when considering bees, setting aside one large area may be more effective at protecting them in the long term; in other words, despite still being in a protected area, the bees inside the three new units may also be affected by this change in monument structuring.

A number of bee species formerly afforded certain levels of protection associated with national monument boundaries are now excluded. In fact, though the lands excluded from the new monuments represent 47% of the total area, they house almost as many bee species as all of the new units combined. This is because many of the most prolific bee study sites along Hole-in-the-Rock Road are now excluded from the units (Fig. 1B). In addition, 84 species are now completely excluded from the monuments. It is possible that some of these excluded species do occur somewhere within the new boundaries, but have not been collected there yet (although it is doubtful that is the case for all of them). Among the excluded bees are many that may prove of great interest to scientists, as they do not fit with any known bee descriptions, and may be new species or subspecies. Also, on that list are bees who reach the northern or southern extent of their range in this part of the world. These populations representing the periphery of their species’ ranges are of particular importance as they can provide valuable information about how bee species might respond to climate change (Kerr et al., 2015). Furthermore, research has illustrated the importance of protecting populations, like those of the newly excluded bee species which are at the edge of their range, as these populations may house genetically, ecologically, or morphologically distinct organisms (e.g., Lesica & Allendorf, 1995; Hardie & Hutchings, 2010; Abeli, Vamosi & Orsenigo, 2018; Papuga et al., 2018).

It is unclear what impact the monument boundary changes will have on these excluded species. Few of the bees that are excluded are universally rare, and most are common in other regions of the western United States, though they may represent peripheral populations as described above. None of the excluded bees appear to be narrow specialists on endemic plants of the Colorado Plateau, either. Bees perform a critical ecological service as pollinators and the loss of bee diversity and their connections to pollination networks can have negative impacts on wild plant populations, as well as ecosystem stability and crop production (e.g., Potts et al., 2010; Carman & Jenkins, 2016). However, the role of these specific bees in maintaining functioning plant–pollinator networks has not been evaluated to any extent. As many of them are hot desert-adapted bees, associated with hot desert-adapted plants, the importance of each in the maintenance of the other in this unique habitat should be evaluated.

Though none of the bees here appear to be currently imperiled, the original GSENM did serve as an important safe harbor for bees, some of which may be experiencing declines in the face of significant urban development elsewhere (Banaszak-Cibicka & Zmihorski, 2012), which can lead to changes in plant–pollinator interactions (Geslin et al., 2013). The minimization of this monument opens the door to further development, such as paving, mining, natural gas extraction, and increased human activity and traffic, and reduces the role these monuments can play in protecting unique pollinators and pollinator communities. What’s more, scientific research into the needs, population dynamics, and identities of these bees has been deprioritized during a time when understanding the role of each specific pollinator is imperative (Allen-Wardell et al., 1998; Potts et al., 2010). In order to minimize the impact of this monument downsizing, we therefore suggest that land managers commit to prioritizing pollinators in current and future land management plans for the monument.

##  Supplemental Information

10.7717/peerj.6057/supp-1 Table S1 A list of all identified bee specimens collected in the lands formerly designated as Grand Staircase-Escalante National Monument Raw Data showing the number of specimens of each species for each of the three new units (Grand Staircase, Kaiparowits, Escalante Canyons) as well as the areas now excluded from monument boundaries. Bees are arranged by family, subfamily, genus, subgenus, and finally species. New species (n. sp.) are those that differ from all published keys and are unique after comparison with all specimens located at the US National Pollinating Insects Laboratory (a collection of nearly 2 million specimens). They are listed with their closest affiliation. Species that do not match with specimen descriptions exactly, nor with known specimens, but are not entirely distinct may represent either variants or new species. We have conservatively listed these as ‘sp.’, rather than as new species, with the closest affiliation in parentheses. *Agapostemon angelicus and A. texanus* females are impossible to distinguish. The number of specimens reported for each of these two species is for the males only, which can be identified.

10.7717/peerj.6057/supp-2 Table S2 Bee species no longer found in newly modified monument boundaries but formerly known from Grand Staircase Escalante National Monument Bee species no longer found in newly modified monument boundaries but formerly known from Grand Staircase Escalante National Monument. Distributions and other biological notes taken from Discoverlife.org (Ascher & Pickering, 2018).

## Additional Information and Declarations

### Data Availability

The following information was supplied regarding data availability: The bees found in each monument and excluded lands are listed in Table S1. Species names and abundance data are provided.
