# Supplementary material for: Reducing protected lands in a hotspot of bee biodiversity: bees of Grand Staircase-Escalante National Monument
Source: PeerJ. 2018 Dec 4;6:e6057. doi: 10.7717/peerj.6057 (PMC6284448; doi:10.7717/peerj.6057)
Supplement: Table S1 — Raw Data showing the number of specimens of each species for each of the three new units (Grand Staircase, Kaiparowits, Escalante Canyons) as well as the areas now excluded from monument boundaries. Bees are arranged by family, subfamily, genus, subgenus, and finally species. New species (n. sp.) are those that differ from all published keys and are unique after comparison with all specimens located at the US National Pollinating Insects Laboratory (a collection of nearly 2 million specimens). They are listed with their closest affiliation. Species that do not match with specimen descriptions exactly, nor with known specimens, but are not entirely distinct may represent either variants or new species. We have conservatively listed these as ‘sp.’, rather than as new species, with the closest affiliation in parentheses. Agapostemon angelicus and A. texanus females are impossible to distinguish. The number of specimens reported for each of these two species is for the males only, which can be identified. [file peerj-06-6057-s001.docx]

**Table S1**

A list of all identified bee specimens collected in the lands formerly designated as Grand Staircase-Escalante National Monument. The number of specimens of each species are reported for each of the three new units (Grand Staircase, Kaiparowits, Escalante Canyons) as well as the areas now excluded from monument boundaries. Bees are arranged by family, subfamily, genus, subgenus, and finally species. New species (n. sp.) are those that differ from all published keys and are unique after comparison with all specimens located at the U.S. National Pollinating Insects Laboratory (a collection of nearly 2 million specimens). They are listed with their closest affiliation. Species that do not match with specimen descriptions exactly, nor with known specimens, but are not entirely distinct may represent either variants or new species. We have conservatively listed these as ‘sp.’, rather than as new species, with the closest affiliation in parentheses. *Agapostemon angelicus and A. texanus* females are impossible to distinguish. The number of specimens reported for each of these two species is for the males only, which can be identified.

| **Species** | **All 3 new monuments combined** | **Escalante Canyons**  **Unit** | **Grand Staircase**  **Unit** | **Kaiparowits**  **Unit** | **Excluded areas** |
| --- | --- | --- | --- | --- | --- |
| Ancylandrena timberlakei | 0 | 0 | 0 | 0 | 1 |
| Andrena (Andrena) frigida | 28 | 3 | 0 | 12 | 0 |
| Andrena (Andrena) macoupinensis | 100 | 19 | 0 | 18 | 1 |
| Andrena (Andrena) milwaukeensis | 1348 | 3 | 40 | 942 | 2 |
| Andrena (Andrena) schuhi | 1072 | 0 | 44 | 738 | 21 |
| Andrena (Belandrena) sphaeralceae | 52 | 3 | 0 | 30 | 1 |
| Andrena (Callandrena) accepta | 4 | 1 | 0 | 0 | 5 |
| Andrena (Callandrena) haynesi | 0 | 0 | 0 | 0 | 3 |
| Andrena (Callandrena) helianthi | 0 | 0 | 0 | 0 | 5 |
| Andrena (Callandrena) n. sp. | 48 | 10 | 2 | 3 | 13 |
| Andrena (Callandrena) neomexicana | 8 | 2 | 0 | 0 | 8 |
| Andrena (Callandrena) pecosana | 8 | 2 | 0 | 0 | 12 |
| Andrena (Callandrena) sp. 1 (aff. pecosana) | 20 | 3 | 0 | 6 | 3 |
| Andrena (Callandrena) sp. 2 (aff. simulata) | 4 | 0 | 0 | 3 | 0 |
| Andrena (Callandrena) utahensis | 316 | 41 | 36 | 60 | 33 |
| Andrena (Callandrena) vulpicolor | 60 | 15 | 0 | 0 | 10 |
| Andrena (Cnemidandrena) costillensis | 192 | 1 | 18 | 114 | 18 |
| Andrena (Cnemidandrena) n. sp. 2 (aff. costillensis) | 84 | 19 | 4 | 0 | 0 |
| Andrena (Cnemidandrena) ramaleyi | 372 | 0 | 18 | 252 | 14 |
| Andrena (Cnemidandrena) sp. 3 (aff. aurihirta) | 272 | 41 | 28 | 39 | 23 |
| Andrena (Cnemidandrena) sp. 4 (aff. colletina) | 136 | 22 | 16 | 12 | 29 |
| Andrena (Conandrena) cheyennorum | 336 | 0 | 0 | 252 | 0 |
| Andrena (Dactylandrena) porterae | 0 | 0 | 0 | 0 | 2 |
| Andrena (Dasyandrena) cristata | 20 | 0 | 2 | 12 | 0 |
| Andrena (Diandrena) evoluta | 1316 | 6 | 0 | 969 | 5 |
| Andrena (Euandrena) astragali | 72 | 0 | 2 | 51 | 0 |
| Andrena (Euandrena) chlorura | 92 | 0 | 42 | 6 | 0 |
| Andrena (Euandrena) lawrencei | 44 | 0 | 0 | 33 | 0 |
| Andrena (Euandrena) nigrihirta | 52 | 0 | 0 | 39 | 0 |
| Andrena (Euandrena) sp. 5 (aff. auricoma) | 4 | 1 | 0 | 0 | 0 |
| Andrena (Euandrena) sp. 6 (aff. nigrihirta) | 12 | 0 | 0 | 9 | 0 |
| Andrena (Holandrena) cressonii | 72 | 0 | 4 | 48 | 30 |
| Andrena (Leucandrena) barbilabris | 68 | 0 | 34 | 0 | 0 |
| Andrena (Melandrena) cerasifolii | 164 | 23 | 0 | 54 | 3 |
| Andrena (Melandrena) lupinorum | 4176 | 1 | 44 | 3063 | 38 |
| Andrena (Melandrena) sola | 8 | 0 | 4 | 0 | 5 |
| Andrena (Melandrena) transnigra | 88 | 1 | 42 | 0 | 6 |
| Andrena (Micrandrena) illinoiensis | 28 | 0 | 0 | 21 | 0 |
| Andrena (Micrandrena) kristina | 28 | 2 | 4 | 9 | 14 |
| Andrena (Micrandrena) melanochroa | 0 | 0 | 0 | 0 | 2 |
| Andrena (Micrandrena) piperi | 164 | 0 | 76 | 9 | 10 |
| Andrena (Onagrandrena) anograe | 84 | 1 | 10 | 45 | 9 |
| Andrena (Onagrandrena) linsleyana | 60 | 14 | 0 | 3 | 11 |
| Andrena (Parandrena) andrenoides | 332 | 36 | 0 | 141 | 1 |
| Andrena (Parandrena) papagorum | 8 | 0 | 0 | 6 | 1 |
| Andrena (Parandrena) wellesleyana | 128 | 32 | 0 | 0 | 0 |
| Andrena (Plastandrena) prunorum | 1704 | 95 | 104 | 837 | 108 |
| Andrena (Rhaphandrena) prima | 0 | 0 | 0 | 0 | 9 |
| Andrena (Scaphandrena) bruneri | 296 | 29 | 32 | 87 | 8 |
| Andrena (Scaphandrena) chapmanae | 116 | 0 | 58 | 0 | 2 |
| Andrena (Scaphandrena) cruciferarum | 0 | 0 | 0 | 0 | 1 |
| Andrena (Scaphandrena) hicksi | 8 | 0 | 0 | 6 | 0 |
| Andrena (Scaphandrena) kaibabensis | 184 | 0 | 2 | 135 | 1 |
| Andrena (Scaphandrena) merriami | 48 | 1 | 16 | 9 | 1 |
| Andrena (Scaphandrena) montrosensis | 16 | 0 | 0 | 12 | 8 |
| Andrena (Scaphandrena) n. sp. (aff. kaibabensis) | 64 | 0 | 30 | 3 | 0 |
| Andrena (Scaphandrena) nigricula | 228 | 15 | 16 | 102 | 23 |
| Andrena (Scaphandrena) scurra | 136 | 1 | 36 | 45 | 6 |
| Andrena (Scaphandrena) sieverti | 0 | 0 | 0 | 0 | 6 |
| Andrena (Scrapteropsis) imitatrix | 4 | 0 | 2 | 0 | 0 |
| Andrena (Simandrena) angustitarsata | 52 | 0 | 26 | 0 | 0 |
| Andrena (Thysandrena) medionitens | 1644 | 3 | 30 | 1179 | 2 |
| Andrena (Thysandrena) sp. 7 (aff. medionitens) | 56 | 0 | 22 | 9 | 3 |
| Andrena (Thysandrena) sp. 8 (aff. w-scripta) | 24 | 0 | 0 | 18 | 4 |
| Andrena (Thysandrena) w-scripta | 600 | 26 | 110 | 207 | 28 |
| Andrena (Trachandrena) cupreotincta | 1608 | 0 | 118 | 1029 | 16 |
| Andrena (Trachandrena) forbesii | 4 | 0 | 2 | 0 | 0 |
| Andrena (Trachandrena) salicifloris | 0 | 0 | 0 | 0 | 1 |
| Andrena (Trachandrena) striatifrons | 128 | 31 | 0 | 3 | 0 |
| Andrena (Trachandrena) zionensis | 584 | 18 | 64 | 288 | 88 |
| Andrena (Tylandrena) hallii | 108 | 3 | 28 | 30 | 5 |
| Andrena (Tylandrena) subaustralis | 208 | 47 | 0 | 15 | 0 |
| Calliopsis (Calliopsima) chlorops | 372 | 11 | 10 | 231 | 23 |
| Calliopsis (Calliopsima) philiphunteri | 20 | 1 | 0 | 12 | 27 |
| Calliopsis (Nomadopsis) puellae | 0 | 0 | 0 | 0 | 35 |
| Calliopsis (Nomadopsis) timberlakei | 28 | 2 | 2 | 12 | 12 |
| Macrotera (Macroterella) opacella | 128 | 22 | 0 | 30 | 29 |
| Macrotera (Macroteropsis) arcuata | 0 | 0 | 0 | 0 | 1 |
| Macrotera (Macroteropsis) latior | 0 | 0 | 0 | 0 | 1 |
| Macrotera (Macroteropsis) n.sp. (aff. portalis) | 0 | 0 | 0 | 0 | 3 |
| Panurginus sp. 1 | 0 | 0 | 0 | 0 | 1 |
| Panurginus sp. 2 | 0 | 0 | 0 | 0 | 6 |
| Perdita sp. E10 | 0 | 0 | 0 | 0 | 5 |
| Perdita sp. E11 | 4 | 0 | 0 | 3 | 0 |
| Perdita sp. E12 | 4 | 0 | 2 | 0 | 1 |
| Perdita sp. E13 | 20 | 0 | 0 | 15 | 0 |
| Perdita sp. E2 | 12 | 3 | 0 | 0 | 6 |
| Perdita sp. E3 | 4 | 0 | 0 | 3 | 1 |
| Perdita sp. E4 | 60 | 14 | 0 | 3 | 10 |
| Perdita sp. E5 | 0 | 0 | 0 | 0 | 7 |
| Perdita sp. E8 | 0 | 0 | 0 | 0 | 1 |
| Perdita sp. E9 | 0 | 0 | 0 | 0 | 27 |
| Perdita (Allomacrotera) moabensis | 60 | 14 | 2 | 0 | 21 |
| Perdita (Cockerellia) albipennis | 48 | 2 | 0 | 30 | 69 |
| Perdita (Cockerellia) coreopsidis | 4 | 1 | 0 | 0 | 0 |
| Perdita (Cockerellia) imbellis | 176 | 12 | 6 | 87 | 30 |
| Perdita (Cockerellia) lingualis | 0 | 0 | 0 | 0 | 3 |
| Perdita (Cockerellia) perpulchra | 276 | 67 | 2 | 3 | 52 |
| Perdita (Cockerellia) verbesinae | 20 | 3 | 0 | 6 | 7 |
| Perdita (Epimacrotera) crassula | 0 | 0 | 0 | 0 | 2 |
| Perdita (Glossoperdita) n. sp. 14 (aff. giliae) | 4 | 1 | 0 | 0 | 163 |
| Perdita (Hexaperdita) asteris | 320 | 80 | 0 | 0 | 1 |
| Perdita (Hexaperdita) heterothecae | 4 | 0 | 0 | 3 | 17 |
| Perdita (Perdita) aridella | 2152 | 320 | 140 | 444 | 557 |
| Perdita (Perdita) calloleuca | 13839 | 4085 | 452 | 558 | 1362 |
| Perdita (Perdita) croceipes | 0 | 0 | 0 | 0 | 11 |
| Perdita (Perdita) crotonis | 0 | 0 | 0 | 0 | 1 |
| Perdita (Perdita) depressa | 279 | 71 | 4 | 60 | 40 |
| Perdita (Perdita) dilecta | 24 | 7 | 0 | 3 | 46 |
| Perdita (Perdita) dubia | 18 | 2 | 0 | 12 | 114 |
| Perdita (Perdita) electa | 120 | 31 | 16 | 3 | 27 |
| Perdita (Perdita) euzonata | 0 | 0 | 0 | 0 | 6 |
| Perdita (Perdita) fallax | 750 | 44 | 62 | 525 | 50 |
| Perdita (Perdita) festiva | 2691 | 2 | 0 | 2685 | 1 |
| Perdita (Perdita) glabrescens | 0 | 0 | 0 | 0 | 1 |
| Perdita (Perdita) hirsuta | 9 | 3 | 0 | 0 | 0 |
| Perdita (Perdita) holoxantha | 27 | 0 | 0 | 27 | 2 |
| Perdita (Perdita) idonea | 108 | 32 | 4 | 6 | 20 |
| Perdita (Perdita) knowltoni | 138 | 32 | 10 | 27 | 222 |
| Perdita (Perdita) lepidosparti | 18 | 0 | 12 | 0 | 1 |
| Perdita (Perdita) luteola | 846 | 244 | 32 | 66 | 101 |
| Perdita (Perdita) mesillensis | 27 | 8 | 2 | 0 | 14 |
| Perdita (Perdita) morula | 318 | 52 | 6 | 153 | 94 |
| Perdita (Perdita) munda | 39 | 1 | 0 | 36 | 48 |
| Perdita (Perdita) n. sp. 1 | 756 | 251 | 0 | 3 | 18 |
| Perdita (Perdita) n. sp. 10 (aff. eremica) | 27 | 0 | 0 | 27 | 183 |
| Perdita (Perdita) n. sp. 11 (aff. elongaticeps) | 3 | 1 | 0 | 0 | 0 |
| Perdita (Perdita) n. sp. 12 (aff. cuspidata) | 0 | 0 | 0 | 0 | 13 |
| Perdita (Perdita) n. sp. 13 (aff. confusa) | 471 | 157 | 0 | 0 | 0 |
| Perdita (Perdita) n. sp. 14 (aff. zonalis) | 36 | 9 | 0 | 9 | 0 |
| Perdita (Perdita) n. sp. 15 | 3 | 1 | 0 | 0 | 102 |
| Perdita (Perdita) n. sp. 2 (aff. zebrata) | 399 | 129 | 0 | 12 | 33 |
| Perdita (Perdita) n. sp. 3 (aff. tortifoliae) | 0 | 0 | 0 | 0 | 1 |
| Perdita (Perdita) n. sp. 4 (aff. subfasciata) | 3 | 0 | 0 | 3 | 144 |
| Perdita (Perdita) n. sp. 5 (aff. sphaeralceae) | 12 | 4 | 0 | 0 | 11 |
| Perdita (Perdita) n. sp. 6 (aff. luteola) | 3 | 1 | 0 | 0 | 0 |
| Perdita (Perdita) n. sp. 7 (aff. laticincta) | 3 | 1 | 0 | 0 | 5 |
| Perdita (Perdita) n. sp. 8 (aff. laticincta) | 6 | 2 | 0 | 0 | 0 |
| Perdita (Perdita) n. sp. 9 (aff. gerhardi) | 6 | 0 | 0 | 6 | 0 |
| Perdita (Perdita) nasuta | 15 | 5 | 0 | 0 | 50 |
| Perdita (Perdita) nuda | 177 | 45 | 2 | 39 | 140 |
| Perdita (Perdita) phymatae | 1494 | 342 | 54 | 387 | 208 |
| Perdita (Perdita) rectangulata | 36 | 1 | 2 | 30 | 103 |
| Perdita (Perdita) salicis | 873 | 236 | 84 | 39 | 1 |
| Perdita (Perdita) similis | 2403 | 392 | 168 | 975 | 732 |
| Perdita (Perdita) sp. 10 (aff. aridella) | 255 | 63 | 10 | 51 | 337 |
| Perdita (Perdita) sp. 11 (aff. apacheorum) | 123 | 0 | 2 | 120 | 0 |
| Perdita (Perdita) sp. 2 (aff. xanthochroa) | 0 | 0 | 0 | 0 | 7 |
| Perdita (Perdita) sp. 3 (aff. varleyi) | 3 | 0 | 0 | 3 | 0 |
| Perdita (Perdita) sp. 4 (aff. punctifera) | 0 | 0 | 0 | 0 | 5 |
| Perdita (Perdita) sp. 5 (aff. munda) | 192 | 0 | 14 | 171 | 39 |
| Perdita (Perdita) sp. 6 (aff. luteiventris) | 39 | 0 | 0 | 39 | 0 |
| Perdita (Perdita) sp. 7 (aff. lateralis) | 48 | 1 | 0 | 45 | 17 |
| Perdita (Perdita) sp. 8 (aff. koebelei) | 0 | 0 | 0 | 0 | 3 |
| Perdita (Perdita) sp. 9 (aff. fuscipes) | 9 | 1 | 0 | 6 | 32 |
| Perdita (Perdita) sp. E1 | 111 | 21 | 0 | 48 | 134 |
| Perdita (Perdita) subfasciata | 4416 | 908 | 152 | 1464 | 1145 |
| Perdita (Perdita) tortifoliae | 567 | 185 | 2 | 9 | 161 |
| Perdita (Perdita) vestita | 321 | 37 | 66 | 111 | 156 |
| Perdita (Perdita) wilmattae | 9 | 0 | 0 | 9 | 15 |
| Perdita (Perdita) xanthochroa | 441 | 126 | 22 | 30 | 51 |
| Perdita (Perdita) xerophila | 87 | 0 | 0 | 87 | 35 |
| Perdita (Perdita) zebrata | 5940 | 1226 | 1130 | 567 | 768 |
| Perdita (Procockerellia) albonotata | 9 | 1 | 4 | 0 | 23 |
| Perdita (Pygoperdita) duplonotata | 624 | 13 | 86 | 456 | 319 |
| Perdita (Pygoperdita) fallugiae | 0 | 0 | 0 | 0 | 11 |
| Perdita (Pygoperdita) mormonica | 9 | 0 | 0 | 9 | 0 |
| Perdita (Pygoperdita) sp. 1 (aff. mohavensis) | 0 | 0 | 0 | 0 | 7 |
| Perdita (Xeromacrotera) cephalotes | 3 | 1 | 0 | 0 | 251 |
| Pseudopanugus (Heterosarus) bakeri | 3 | 1 | 0 | 0 | 0 |
| Pseudopanugus (Heterosarus) n. sp. | 129 | 2 | 8 | 111 | 21 |
| Pseudopanugus (Heterosarus) sp. E1 | 18 | 3 | 4 | 3 | 0 |
| Pseudopanugus (Heterosarus) sp. E2 | 33 | 0 | 18 | 6 | 0 |
| Pseudopanugus (Heterosarus) sp. E3 | 18 | 0 | 0 | 18 | 56 |
| Pseudopanugus (Pterosarus) irregularis | 3 | 1 | 0 | 0 | 157 |
| Pseudopanugus (Pterosarus) n.sp. (aff. irregularis) | 1110 | 77 | 0 | 879 | 7 |
| Pseudopanugus (Pterosarus) sp. 1 (aff. porterae) | 3 | 0 | 0 | 3 | 0 |
| Pseudopanugus (Pterosarus) sp. E1 | 24 | 2 | 6 | 9 | 28 |
| Pseudopanugus (Pterosarus) sp. E2 | 18 | 0 | 10 | 3 | 0 |
| Pseudopanugus (Pterosarus) sp. E3 | 3 | 0 | 0 | 3 | 35 |
| Pseudopanugus (Pterosarus) sp. E4 | 0 | 0 | 0 | 0 | 1 |
| Anthophora (Anthophoroides) n. sp. 1 | 6 | 2 | 0 | 0 | 5 |
| Anthophora (Anthophoroides) n. sp. 2 | 33 | 10 | 0 | 3 | 103 |
| Anthophora (Anthophoroides) pueblo | 0 | 0 | 0 | 0 | 2 |
| Anthophora (Heliophila) albata | 732 | 210 | 24 | 66 | 27 |
| Anthophora (Lophanthophora) affabilis | 57 | 5 | 2 | 39 | 20 |
| Anthophora (Lophanthophora) dammersi | 30 | 0 | 0 | 30 | 16 |
| Anthophora (Lophanthophora) neglecta | 303 | 39 | 8 | 174 | 29 |
| Anthophora (Lophanthophora) pacifica | 75 | 0 | 4 | 69 | 16 |
| Anthophora (Lophanthophora) porterae | 33 | 3 | 0 | 24 | 8 |
| Anthophora (Lophanthophora) ursina | 66 | 0 | 2 | 63 | 4 |
| Anthophora (Melea) bomboides | 156 | 26 | 2 | 75 | 5 |
| Anthophora (Melea) occidentalis | 9 | 0 | 0 | 9 | 56 |
| Anthophora (Micranthophora) albata | 6 | 2 | 0 | 0 | 0 |
| Anthophora (Micranthophora) curta | 3 | 0 | 0 | 3 | 6 |
| Anthophora (Micranthophora) escalante | 3 | 0 | 2 | 0 | 3 |
| Anthophora (Micranthophora) peritomae | 969 | 189 | 98 | 255 | 67 |
| Anthophora (Micranthophora) petrophila | 1251 | 156 | 72 | 655 | 330 |
| Anthophora (Mystacanthophora) montana | 48 | 1 | 0 | 30 | 10 |
| Anthophora (Mystacanthophora) urbana | 2877 | 292 | 636 | 698 | 367 |
| Anthophora (Pyganthophora) edwardsii | 0 | 0 | 0 | 0 | 1 |
| Anthophora (Pyganthophora) lesquerellae | 117 | 24 | 2 | 28 | 42 |
| Anthophora (Pyganthophora) n. sp. | 48 | 14 | 0 | 4 | 1 |
| Anthophorula (Anthophorula) albata | 0 | 0 | 0 | 0 | 30 |
| Anthophorula (Anthophorula) crenulata | 0 | 0 | 0 | 0 | 38 |
| Apis mellifera | 6168 | 1165 | 684 | 1098 | 418 |
| Bombus (Bombias) nevadensis | 33 | 0 | 0 | 22 | 0 |
| Bombus (Bombus) occidentalis | 18 | 1 | 0 | 10 | 0 |
| Bombus (Fervidobombus) fervidus | 81 | 9 | 20 | 16 | 0 |
| Bombus (Pyrobombus) huntii | 393 | 44 | 0 | 174 | 52 |
| Bombus (Pyrobombus) melanopygus | 147 | 2 | 0 | 94 | 0 |
| Bombus (Separatobombus) morrisoni | 1200 | 161 | 18 | 460 | 92 |
| Ceratina (Zadontomerus) apacheorum | 105 | 17 | 8 | 28 | 230 |
| Ceratina (Zadontomerus) nanula | 14208 | 2151 | 728 | 4442 | 452 |
| Ceratina (Zadontomerus) neomexicana | 1632 | 5 | 14 | 1064 | 64 |
| Ceratina (Zadontomerus) pacifica | 1583 | 305 | 258 | 200 | 144 |
| Diadasia australis | 130 | 17 | 6 | 90 | 101 |
| Diadasia diminuta | 846 | 202 | 262 | 180 | 176 |
| Diadasia enavata | 10 | 1 | 2 | 6 | 47 |
| Diadasia lutzi | 138 | 60 | 0 | 18 | 66 |
| Diadasia martialis | 2 | 0 | 0 | 2 | 9 |
| Diadasia ochracea | 8 | 4 | 0 | 0 | 12 |
| Diadasia rinconis | 10 | 1 | 2 | 6 | 6 |
| Diadasia vallicola | 0 | 0 | 0 | 0 | 1 |
| Epeolus mesillae | 2 | 0 | 2 | 0 | 5 |
| Epeolus minimus | 94 | 3 | 0 | 88 | 9 |
| Epeolus pusillus | 66 | 25 | 10 | 6 | 4 |
| Epeolus scutellaris | 8 | 4 | 0 | 0 | 0 |
| Epeolus sp. 3 | 0 | 0 | 0 | 0 | 1 |
| Eucera (Synhalonia) acerba | 14 | 0 | 0 | 14 | 1 |
| Eucera (Synhalonia) edwardsii | 6 | 0 | 6 | 0 | 1 |
| Eucera (Synhalonia) frater | 254 | 0 | 26 | 228 | 1 |
| Eucera (Synhalonia) fulvitarsis | 300 | 1 | 24 | 274 | 10 |
| Eucera (Synhalonia) lunata | 8 | 0 | 8 | 0 | 2 |
| Eucera (Synhalonia) mohavensis | 34 | 12 | 0 | 10 | 27 |
| Eucera (Synhalonia) phaceliae | 112 | 55 | 2 | 0 | 15 |
| Eucera (Synhalonia) primaveris | 32 | 0 | 0 | 32 | 18 |
| Eucera (Synhalonia) quadricincta | 8 | 0 | 0 | 8 | 2 |
| Eucera (Synhalonia) speciosa | 6 | 2 | 0 | 2 | 5 |
| Eucera (Synhalonia) territella | 106 | 21 | 12 | 52 | 34 |
| Exomalopsis (Phanomalopsis) solidaginis | 308 | 147 | 0 | 14 | 90 |
| Habropoda cineraria | 138 | 0 | 10 | 128 | 8 |
| Habropoda excellens | 60 | 22 | 6 | 10 | 13 |
| Habropoda morrisoni | 334 | 28 | 30 | 248 | 69 |
| Hexepeolus rhodogyne | 0 | 0 | 0 | 0 | 1 |
| Holcopasites pulchellus | 2 | 1 | 0 | 0 | 7 |
| Melecta (Melecta) alexanderi | 0 | 0 | 0 | 0 | 1 |
| Melecta (Melecta) bohartorum | 0 | 0 | 0 | 0 | 2 |
| Melecta (Melecta) pacifica | 6 | 0 | 0 | 6 | 0 |
| Melecta (Melecta) separata | 4 | 1 | 0 | 2 | 2 |
| Melecta (Melecta) thoracica | 116 | 2 | 4 | 108 | 15 |
| Melissodes sp. E1 | 2 | 0 | 0 | 2 | 5 |
| Melissodes sp. m1 | 44 | 2 | 2 | 38 | 21 |
| Melissodes (Callimelissodes) coloradensis | 0 | 0 | 0 | 0 | 2 |
| Melissodes (Callimelissodes) compositus | 0 | 0 | 0 | 0 | 1 |
| Melissodes (Callimelissodes) glenwoodensis | 214 | 59 | 32 | 64 | 65 |
| Melissodes (Eumelissodes) agilis | 128 | 42 | 2 | 42 | 29 |
| Melissodes (Eumelissodes) bicoloratus | 72 | 36 | 0 | 0 | 2 |
| Melissodes (Eumelissodes) bimatris | 494 | 102 | 132 | 158 | 131 |
| Melissodes (Eumelissodes) brevipyga | 0 | 0 | 0 | 0 | 2 |
| Melissodes (Eumelissodes) grindeliae | 342 | 0 | 4 | 338 | 0 |
| Melissodes (Eumelissodes) illatus | 0 | 0 | 0 | 0 | 1 |
| Melissodes (Eumelissodes) lutulentus | 132 | 0 | 44 | 88 | 13 |
| Melissodes (Eumelissodes) menuachus | 20 | 5 | 4 | 6 | 7 |
| Melissodes (Eumelissodes) montanus | 18 | 0 | 0 | 18 | 0 |
| Melissodes (Eumelissodes) pallidisignatus | 114 | 19 | 38 | 38 | 48 |
| Melissodes (Eumelissodes) perlusus | 64 | 26 | 0 | 12 | 16 |
| Melissodes (Eumelissodes) perpolitus | 48 | 13 | 4 | 18 | 39 |
| Melissodes (Eumelissodes) rustica | 708 | 0 | 0 | 708 | 3 |
| Melissodes (Eumelissodes) saponellus | 18 | 9 | 0 | 0 | 19 |
| Melissodes (Eumelissodes) semilupinus | 160 | 53 | 28 | 26 | 53 |
| Melissodes (Eumelissodes) snowii | 14 | 7 | 0 | 0 | 2 |
| Melissodes (Eumelissodes) subagilis | 8 | 0 | 0 | 8 | 0 |
| Melissodes (Eumelissodes) submenuacha | 4 | 0 | 2 | 2 | 2 |
| Melissodes (Eumelissodes) tristis | 524 | 92 | 46 | 294 | 233 |
| Melissodes (Eumelissodes) utahensis | 116 | 15 | 36 | 50 | 64 |
| Melissodes (Eumelissodes) verbesinarum | 38 | 13 | 0 | 12 | 26 |
| Melissodes (Tachymelissodes) dagosus | 8 | 0 | 0 | 8 | 1 |
| Neolarra (Neolarra) penicula | 2 | 0 | 0 | 2 | 5 |
| Neolarra (Neolarra) sp. 1 | 28 | 12 | 0 | 4 | 8 |
| Neolarra (Neolarra) verbesinae | 0 | 0 | 0 | 0 | 7 |
| Neolarra (Phileremulus) cockerelli | 6 | 3 | 0 | 0 | 8 |
| Neolarra (Phileremulus) vigilans | 4 | 2 | 0 | 0 | 6 |
| Nomada (Centrias) crotchii | 30 | 5 | 14 | 6 | 3 |
| Nomada (Centrias) munda | 2 | 0 | 2 | 0 | 1 |
| Nomada (Centrias) sp. E1 | 12 | 4 | 4 | 0 | 7 |
| Nomada (Centrias) sp. E2 | 0 | 0 | 0 | 0 | 8 |
| Nomada (Holonomada) edwardsii | 78 | 0 | 6 | 72 | 0 |
| Nomada (Holonomada) parkeri | 12 | 0 | 0 | 12 | 3 |
| Nomada (Laminomada) n. sp. | 4 | 0 | 0 | 4 | 1 |
| Nomada (Micronomada) sp. E1 | 2 | 1 | 0 | 0 | 3 |
| Nomada (Micronomada) sp. E2 | 8 | 4 | 0 | 0 | 0 |
| Nomada (Micronomada) sp. E3 | 2 | 1 | 0 | 0 | 0 |
| Nomada (Micronomada) sp. E4 | 4 | 2 | 0 | 0 | 3 |
| Nomada (Nomada) sp. E1 | 308 | 4 | 96 | 204 | 7 |
| Nomada (Nomada) sp. E10 | 2 | 0 | 0 | 2 | 1 |
| Nomada (Nomada) sp. E11 | 46 | 2 | 12 | 30 | 5 |
| Nomada (Nomada) sp. E12 | 14 | 0 | 8 | 6 | 1 |
| Nomada (Nomada) sp. E13 | 0 | 0 | 0 | 0 | 1 |
| Nomada (Nomada) sp. E14 | 14 | 1 | 10 | 2 | 0 |
| Nomada (Nomada) sp. E15 | 34 | 0 | 0 | 34 | 1 |
| Nomada (Nomada) sp. E16 | 28 | 2 | 2 | 22 | 0 |
| Nomada (Nomada) sp. E17 | 4 | 0 | 2 | 2 | 2 |
| Nomada (Nomada) sp. E18 | 52 | 0 | 2 | 50 | 0 |
| Nomada (Nomada) sp. E19 | 6 | 0 | 4 | 2 | 0 |
| Nomada (Nomada) sp. E2 | 34 | 0 | 0 | 34 | 0 |
| Nomada (Nomada) sp. E20 | 2 | 0 | 2 | 0 | 0 |
| Nomada (Nomada) sp. E22 | 14 | 0 | 12 | 2 | 4 |
| Nomada (Nomada) sp. E23 | 0 | 0 | 0 | 0 | 4 |
| Nomada (Nomada) sp. E25 | 0 | 0 | 0 | 0 | 1 |
| Nomada (Nomada) sp. E3 | 236 | 0 | 14 | 222 | 0 |
| Nomada (Nomada) sp. E4 | 286 | 1 | 14 | 270 | 10 |
| Nomada (Nomada) sp. E5 | 12 | 1 | 0 | 10 | 7 |
| Nomada (Nomada) sp. E6 | 8 | 0 | 0 | 8 | 0 |
| Nomada (Nomada) sp. E7 | 42 | 0 | 0 | 42 | 2 |
| Nomada (Nomada) sp. E8 | 72 | 0 | 14 | 58 | 4 |
| Nomada (Nomadita) mutans | 8 | 3 | 2 | 0 | 1 |
| Oreopasites sp. | 2 | 0 | 0 | 2 | 0 |
| Paranomada sp. 1 (aff. nitida) | 2 | 1 | 0 | 0 | 0 |
| Svastra (Epimelissodes) helianthelli | 0 | 0 | 0 | 0 | 6 |
| Svastra (Epimelissodes) obliqua | 2 | 0 | 2 | 0 | 6 |
| Triepeolus balteatus | 16 | 5 | 6 | 0 | 18 |
| Triepeolus dacotensis | 6 | 3 | 0 | 0 | 0 |
| Triepeolus denverensis | 6 | 1 | 2 | 2 | 1 |
| Triepeolus diversipes | 2 | 1 | 0 | 0 | 2 |
| Triepeolus eldoradensis | 32 | 9 | 10 | 4 | 0 |
| Triepeolus helianthi | 0 | 0 | 0 | 0 | 4 |
| Triepeolus micropygius | 2 | 1 | 0 | 0 | 1 |
| Triepeolus morpho sp. 51 | 0 | 0 | 0 | 0 | 1 |
| Triepeolus morpho sp. 69 | 10 | 5 | 0 | 0 | 0 |
| Triepeolus morpho sp. 76 | 2 | 1 | 0 | 0 | 0 |
| Triepeolus norae | 2 | 0 | 0 | 2 | 0 |
| Triepeolus sp. 42 | 8 | 2 | 0 | 4 | 2 |
| Triepeolus subalpinus | 42 | 0 | 0 | 42 | 0 |
| Triepeolus timberlakei | 38 | 13 | 2 | 10 | 15 |
| Xeromelecta (Melectomorpha) californica | 20 | 1 | 6 | 12 | 19 |
| Xylocopa (Notoxylocopa) tabaniformis | 54 | 19 | 2 | 14 | 0 |
| Xylocopa (Xylocopoides) californica | 388 | 108 | 26 | 146 | 5 |
| Zacosmia maculata | 22 | 10 | 0 | 2 | 16 |
| Colletes compactus | 80 | 6 | 8 | 60 | 3 |
| Colletes daleae | 1046 | 267 | 80 | 432 | 164 |
| Colletes eulophi | 120 | 1 | 10 | 108 | 10 |
| Colletes fulgidus | 0 | 0 | 0 | 0 | 1 |
| Colletes gypsicolens | 578 | 162 | 162 | 92 | 127 |
| Colletes intermixtus | 2 | 1 | 0 | 0 | 1 |
| Colletes kincaidii | 2 | 0 | 0 | 2 | 0 |
| Colletes larreae | 2 | 0 | 0 | 2 | 72 |
| Colletes laticinctus | 16 | 6 | 4 | 0 | 25 |
| Colletes louisae | 82 | 27 | 12 | 16 | 224 |
| Colletes lutzi | 12 | 1 | 0 | 10 | 0 |
| Colletes mandibularis | 18 | 8 | 2 | 0 | 0 |
| Colletes n. sp. 1 (aff. algarobiae) | 4 | 2 | 0 | 0 | 0 |
| Colletes n. sp. 2 (aff. aberrans) | 12 | 6 | 0 | 0 | 0 |
| Colletes n. sp. 3 | 4 | 0 | 4 | 0 | 2 |
| Colletes petalostemonis | 280 | 132 | 2 | 14 | 38 |
| Colletes phaceliae | 2012 | 92 | 38 | 1790 | 353 |
| Colletes simulans | 120 | 23 | 20 | 54 | 36 |
| Colletes slevini | 782 | 137 | 60 | 448 | 112 |
| Colletes sp. 1 | 12 | 3 | 4 | 2 | 51 |
| Colletes sp. 2 (aff. petalostemonis) | 34 | 14 | 6 | 0 | 15 |
| Colletes sphaeralceae | 524 | 260 | 2 | 2 | 54 |
| Hylaeus (Hylaeus) granulatus | 12 | 5 | 0 | 2 | 0 |
| Hylaeus (Hylaeus) leptocephalus | 26 | 11 | 0 | 4 | 3 |
| Hylaeus (Hylaeus) mesillae | 972 | 568 | 14 | 30 | 15 |
| Hylaeus (Hylaeus) rudbeckiae | 2 | 0 | 4 | 0 | 0 |
| Hylaeus (Hylaeus) sp. 1 (aff. mesillae) | 195 | 195 | 0 | 0 | 0 |
| Hylaeus (Hylaeus) verticalis | 1 | 0 | 2 | 0 | 0 |
| Hylaeus (Paraprosopis) coloradensis | 2 | 1 | 2 | 0 | 0 |
| Hylaeus (Paraprosopis) megalotis | 683 | 503 | 100 | 260 | 246 |
| Hylaeus (Paraprosopis) n. sp. 1 (aff. cookii) | 628 | 432 | 158 | 234 | 600 |
| Hylaeus (Paraprosopis) n. sp. 2 | 97 | 69 | 12 | 44 | 32 |
| Hylaeus (Paraprosopis) wootoni | 476 | 16 | 116 | 804 | 18 |
| Hylaeus (Prosopis) n. sp. 3 | 98 | 74 | 24 | 24 | 912 |
| Agapostemon (Agapostemon) angelicus | 84 | 67 | 34 | 108 | 147 |
| Agapostemon (Agapostemon) femoratus | 1 | 0 | 0 | 2 | 1 |
| Agapostemon (Agapostemon) melliventris | 6 | 6 | 0 | 0 | 9 |
| Agapostemon (Agapostemon) n. sp. (aff splendens) | 3 | 0 | 4 | 2 | 4 |
| Agapostemon (Agapostemon) texanus | 180 | 51 | 2 | 342 | 5 |
| Dieunomia (Dieunomia) heteropoda | 10 | 10 | 0 | 0 | 1 |
| Dieunomia (Epinomia) nevadensis | 264 | 264 | 0 | 0 | 36 |
| Dufourea contarovici | 2 | 0 | 0 | 4 | 1 |
| Dufourea harveyi | 0 | 0 | 0 | 0 | 1 |
| Dufourea malacothricis | 10 | 1 | 0 | 18 | 128 |
| Dufourea n. sp. 2 | 7 | 0 | 0 | 14 | 1 |
| Dufourea sandhouseae | 20 | 0 | 0 | 40 | 14 |
| Dufourea sp. 1 (aff. saundersi) | 0 | 0 | 0 | 0 | 2 |
| Dufourea tinsleyi | 5 | 0 | 0 | 10 | 0 |
| Halictus (Nealictus) farinosus | 145 | 14 | 20 | 242 | 25 |
| Halictus (Odontalictus) ligatus | 28 | 3 | 2 | 48 | 5 |
| Halictus (Protohalictus) rubicundus | 1 | 0 | 2 | 0 | 11 |
| Halictus (Seladonia) confusus | 8 | 0 | 0 | 16 | 0 |
| Halictus (Seladonia) tripartitus | 3340 | 56 | 150 | 6418 | 336 |
| Lasioglossum (Dialictus) abundipunctum | 183 | 7 | 4 | 348 | 25 |
| Lasioglossum (Dialictus) albohirtum | 412 | 93 | 14 | 624 | 169 |
| Lasioglossum (Dialictus) aliud | 2 | 0 | 0 | 4 | 26 |
| Lasioglossum (Dialictus) brunneiventre | 6 | 6 | 0 | 0 | 1 |
| Lasioglossum (Dialictus) clarissimum | 3 | 3 | 0 | 0 | 6 |
| Lasioglossum (Dialictus) clematisellum | 275 | 174 | 88 | 114 | 282 |
| Lasioglossum (Dialictus) griswoldi | 1 | 0 | 2 | 0 | 0 |
| Lasioglossum (Dialictus) hudsoniellum | 31 | 16 | 2 | 28 | 55 |
| Lasioglossum (Dialictus) hyalinum | 71 | 12 | 0 | 118 | 53 |
| Lasioglossum (Dialictus) impavidum | 20 | 2 | 8 | 28 | 141 |
| Lasioglossum (Dialictus) incompletum | 1 | 0 | 0 | 2 | 0 |
| Lasioglossum (Dialictus) microlepoides | 3 | 1 | 0 | 4 | 5 |
| Lasioglossum (Dialictus) nevadense | 702 | 107 | 100 | 1090 | 190 |
| Lasioglossum (Dialictus) pallidellum | 1 | 0 | 0 | 2 | 21 |
| Lasioglossum (Dialictus) perdifficile | 49 | 7 | 32 | 52 | 62 |
| Lasioglossum (Dialictus) perparvum | 2 | 0 | 0 | 4 | 3 |
| Lasioglossum (Dialictus) petrellum | 44 | 14 | 36 | 24 | 1 |
| Lasioglossum (Dialictus) prasinogaster | 7 | 1 | 2 | 10 | 2 |
| Lasioglossum (Dialictus) pruinosum | 9 | 0 | 16 | 2 | 134 |
| Lasioglossum (Dialictus) ruidosense | 267 | 35 | 32 | 432 | 8 |
| Lasioglossum (Dialictus) sedi | 0 | 0 | 0 | 0 | 1 |
| Lasioglossum (Dialictus) semibrunneum | 0 | 0 | 0 | 0 | 10 |
| Lasioglossum (Dialictus) semicaeruleum | 69 | 9 | 24 | 96 | 77 |
| Lasioglossum (Dialictus) sp. 1 (aff. albuquerquense) | 147 | 128 | 4 | 34 | 17 |
| Lasioglossum (Dialictus) sp. 2 (aff. aliud) | 3 | 1 | 0 | 4 | 26 |
| Lasioglossum (Dialictus) sp. 3 (aff. hudsoniellum) | 2 | 1 | 0 | 2 | 12 |
| Lasioglossum (Dialictus) sp. 4 (aff. hyalinum) | 10 | 2 | 8 | 8 | 11 |
| Lasioglossum (Dialictus) sp. 5 (aff. impavidum) | 69 | 7 | 40 | 84 | 17 |
| Lasioglossum (Dialictus) sp. 6 (aff. pacatum) | 1 | 0 | 0 | 2 | 2 |
| Lasioglossum (Dialictus) sp. 7 (aff. Hunteri) | 5 | 5 | 0 | 0 | 8 |
| Lasioglossum (Dialictus) sp. 8 (aff. nevadense) | 20 | 5 | 6 | 24 | 7 |
| Lasioglossum (Dialictus) sp. 9 (aff. pruinosus) | 2 | 0 | 2 | 2 | 0 |
| Lasioglossum (Dialictus) sp. A | 7 | 2 | 0 | 10 | 50 |
| Lasioglossum (Dialictus) sp. E1 | 55 | 49 | 0 | 12 | 2 |
| Lasioglossum (Dialictus) sp. E10 | 3 | 1 | 0 | 4 | 1 |
| Lasioglossum (Dialictus) sp. E11 | 15 | 3 | 8 | 16 | 23 |
| Lasioglossum (Dialictus) sp. E12 | 1 | 0 | 0 | 2 | 0 |
| Lasioglossum (Dialictus) sp. E14 | 1 | 1 | 0 | 0 | 0 |
| Lasioglossum (Dialictus) sp. E16 | 4 | 1 | 2 | 4 | 1 |
| Lasioglossum (Dialictus) sp. E18 | 4 | 0 | 8 | 0 | 0 |
| Lasioglossum (Dialictus) sp. E19 | 11 | 1 | 0 | 20 | 3 |
| Lasioglossum (Dialictus) sp. E2 | 24 | 5 | 6 | 32 | 10 |
| Lasioglossum (Dialictus) sp. E22 | 47 | 2 | 6 | 84 | 111 |
| Lasioglossum (Dialictus) sp. E23 | 17 | 17 | 0 | 0 | 34 |
| Lasioglossum (Dialictus) sp. E24 | 18 | 7 | 16 | 6 | 34 |
| Lasioglossum (Dialictus) sp. E3 | 2 | 0 | 0 | 4 | 1 |
| Lasioglossum (Dialictus) sp. E5 | 2 | 1 | 0 | 2 | 5 |
| Lasioglossum (Dialictus) sp. E6 | 20 | 14 | 0 | 12 | 32 |
| Lasioglossum (Dialictus) sp. E7 | 4 | 3 | 2 | 0 | 7 |
| Lasioglossum (Dialictus) sp. E8 | 6 | 2 | 8 | 0 | 1 |
| Lasioglossum (Dialictus) sp. E9 | 49 | 29 | 0 | 40 | 10 |
| Lasioglossum (Dialictus) sp. M11 | 1 | 1 | 0 | 0 | 3 |
| Lasioglossum (Dialictus) sp. M15 | 4 | 1 | 6 | 0 | 12 |
| Lasioglossum (Dialictus) sp. M17 | 14 | 7 | 2 | 12 | 38 |
| Lasioglossum (Dialictus) sp. M2 | 5 | 2 | 0 | 6 | 0 |
| Lasioglossum (Dialictus) sp. M3 | 4 | 0 | 2 | 6 | 1 |
| Lasioglossum (Dialictus) tegulare | 83 | 76 | 2 | 12 | 5 |
| Lasioglossum (Dialictus) tegulariforme | 13 | 9 | 0 | 8 | 28 |
| Lasioglossum (Evylaeus) cooleyi | 0 | 0 | 0 | 0 | 2 |
| Lasioglossum (Evylaeus) inconditum | 271 | 3 | 6 | 320 | 14 |
| Lasioglossum (Evylaeus) nigrescens | 11 | 0 | 14 | 4 | 3 |
| Lasioglossum (Evylaeus) ovaliceps | 97 | 11 | 36 | 68 | 15 |
| Lasioglossum (Evylaeus) pectoraloides | 4 | 4 | 0 | 0 | 0 |
| Lasioglossum (Evylaeus) pulveris | 286 | 68 | 26 | 205 | 109 |
| Lasioglossum (Evylaeus) sp. E1 | 49 | 6 | 52 | 17 | 3 |
| Lasioglossum (Evylaeus) sp. E2 | 164 | 36 | 32 | 112 | 16 |
| Lasioglossum (Evylaeus) sp. E3 (aff. nigrescens) | 1 | 0 | 2 | 0 | 0 |
| Lasioglossum (Evylaeus) sp. E5 | 6 | 5 | 2 | 0 | 4 |
| Lasioglossum (Evylaeus) sp. E7 | 13 | 8 | 8 | 1 | 1 |
| Lasioglossum (Evylaeus) swenki | 8 | 1 | 0 | 7 | 13 |
| Lasioglossum (Lasioglossum) egregium | 176 | 17 | 2 | 158 | 3 |
| Lasioglossum (Lasioglossum) heterorhinum | 9 | 0 | 16 | 1 | 3 |
| Lasioglossum (Lasioglossum) lampronotum | 31 | 7 | 4 | 22 | 39 |
| Lasioglossum (Lasioglossum) sisymbrii | 272 | 27 | 18 | 236 | 96 |
| Lasioglossum (Sphecodogastra) lusoria | 24 | 15 | 0 | 9 | 25 |
| Lasioglossum (Sphecodogastra) noctivaga | 2 | 0 | 0 | 2 | 9 |
| Nomia (Acunomia) tetrazonata | 1 | 0 | 0 | 1 | 1 |
| Sphecodes sp. E1 | 11 | 3 | 2 | 7 | 0 |
| Sphecodes sp. E10 | 1 | 0 | 2 | 0 | 0 |
| Sphecodes sp. E11 | 1 | 0 | 0 | 1 | 0 |
| Sphecodes sp. E12 | 6 | 0 | 0 | 6 | 0 |
| Sphecodes sp. E13 | 4 | 4 | 0 | 0 | 0 |
| Sphecodes sp. E14 | 15 | 10 | 0 | 5 | 14 |
| Sphecodes sp. E15 | 5 | 2 | 6 | 0 | 0 |
| Sphecodes sp. E16 | 2 | 0 | 0 | 2 | 0 |
| Sphecodes sp. E17 | 1 | 0 | 0 | 1 | 2 |
| Sphecodes sp. E18 | 9 | 3 | 2 | 5 | 7 |
| Sphecodes sp. E2 | 322 | 3 | 6 | 316 | 1 |
| Sphecodes sp. E3 | 42 | 7 | 10 | 30 | 12 |
| Sphecodes sp. E4 | 2 | 0 | 2 | 1 | 0 |
| Sphecodes sp. E5 | 5 | 0 | 2 | 4 | 0 |
| Sphecodes sp. E6 | 5 | 3 | 2 | 1 | 0 |
| Sphecodes sp. E7 | 42 | 11 | 0 | 31 | 10 |
| Sphecodes sp. E8 | 128 | 34 | 4 | 92 | 50 |
| Sphecodes sp. E9 | 2 | 1 | 0 | 1 | 0 |
| Anthidiellum (Loyalanthidium) notatum | 68 | 41 | 30 | 12 | 21 |
| Anthidium (Anthidium) atripes | 10 | 2 | 4 | 6 | 85 |
| Anthidium (Anthidium) atripoides | 25 | 13 | 0 | 12 | 30 |
| Anthidium (Anthidium) cockerelli | 4 | 2 | 0 | 2 | 30 |
| Anthidium (Anthidium) dammersi | 5 | 4 | 0 | 1 | 22 |
| Anthidium (Anthidium) duomarginatum | 0 | 0 | 0 | 0 | 1 |
| Anthidium (Anthidium) emarginatum | 1 | 1 | 0 | 0 | 0 |
| Anthidium (Anthidium) maculosum | 15 | 4 | 6 | 8 | 6 |
| Anthidium (Anthidium) mormonum | 128 | 37 | 12 | 85 | 21 |
| Anthidium (Anthidium) palmarum | 1 | 0 | 0 | 1 | 5 |
| Anthidium (Anthidium) placitum | 71 | 25 | 22 | 35 | 40 |
| Anthidium (Anthidium) porterae | 1 | 0 | 0 | 1 | 1 |
| Anthidium (Anthidium) schwarzi | 1 | 0 | 2 | 0 | 1 |
| Ashmeadiella (Arogochila) australis | 101 | 91 | 20 | 0 | 6 |
| Ashmeadiella (Arogochila) cazieri | 1 | 1 | 0 | 0 | 11 |
| Ashmeadiella (Arogochila) erema | 4 | 0 | 0 | 4 | 8 |
| Ashmeadiella (Arogochila) lutzi | 2 | 0 | 0 | 2 | 8 |
| Ashmeadiella (Arogochila) n. sp. 1 (aff. leachi) | 0 | 0 | 0 | 0 | 2 |
| Ashmeadiella (Arogochila) n. sp. 2 (aff. micheneri) | 2 | 0 | 0 | 2 | 74 |
| Ashmeadiella (Arogochila) n. sp. 3 (aff. salviae) | 1 | 0 | 2 | 0 | 0 |
| Ashmeadiella (Ashmeadiella) aridula | 589 | 549 | 24 | 28 | 114 |
| Ashmeadiella (Ashmeadiella) bucconis | 369 | 268 | 46 | 78 | 178 |
| Ashmeadiella (Ashmeadiella) cactorum | 484 | 206 | 278 | 139 | 194 |
| Ashmeadiella (Ashmeadiella) californica | 14 | 3 | 8 | 7 | 3 |
| Ashmeadiella (Ashmeadiella) cubiceps | 5 | 4 | 2 | 0 | 5 |
| Ashmeadiella (Ashmeadiella) difugita | 1 | 1 | 0 | 0 | 0 |
| Ashmeadiella (Ashmeadiella) foveata | 9 | 0 | 6 | 6 | 10 |
| Ashmeadiella (Ashmeadiella) gillettei | 21 | 9 | 2 | 11 | 223 |
| Ashmeadiella (Ashmeadiella) meliloti | 91 | 66 | 22 | 14 | 101 |
| Ashmeadiella (Ashmeadiella) opuntiae | 347 | 195 | 30 | 137 | 117 |
| Ashmeadiella (Ashmeadiella) sonora | 13 | 4 | 2 | 8 | 90 |
| Ashmeadiella (Ashmeadiella) sp. 1 (aff. rufipes) | 0 | 0 | 0 | 0 | 4 |
| Ashmeadiella (Ashmeadiella) sp. 2 (aff. titusi) | 0 | 0 | 0 | 0 | 11 |
| Ashmeadiella (Ashmeadiella) vandykiella | 11 | 4 | 6 | 4 | 29 |
| Ashmeadiella (Cubitognatha) xenomastax | 0 | 0 | 0 | 0 | 48 |
| Atoposmia (Atoposmia) n. sp. 1 | 42 | 42 | 0 | 0 | 6 |
| Atoposmia (Atoposmia) n. sp. 2 (aff. anthodyta) | 4 | 1 | 2 | 2 | 7 |
| Atoposmia (Atoposmia) sp. 1 (aff. triodonta) | 4 | 0 | 8 | 0 | 0 |
| Atoposmia (Eremosmia) n. sp. 3 (aff. daleae) | 2 | 0 | 0 | 2 | 45 |
| Coelioxys (Boreocoelioxys) rufitarsis | 3 | 3 | 0 | 0 | 0 |
| Coelioxys (Boreocoelioxys) sayi | 1 | 1 | 0 | 0 | 0 |
| Coelioxys (Coelioxys) hirsutissima | 1 | 0 | 0 | 1 | 1 |
| Coelioxys (Coelioxys) mitchelli | 1 | 1 | 0 | 0 | 0 |
| Coelioxys (Cyrtocoelioxys) gilensis | 0 | 0 | 0 | 0 | 5 |
| Coelioxys (Synocoelioxys) apacheorum | 0 | 0 | 0 | 0 | 1 |
| Coelioxys (Synocoelioxys) hunteri | 9 | 8 | 2 | 0 | 2 |
| Coelioxys (Xerocoelioxys) grindeliae | 9 | 2 | 0 | 7 | 4 |
| Coelioxys (Xerocoelioxys) mesae | 2 | 2 | 0 | 0 | 0 |
| Dianthidium (Adanthidium) arizonicum | 12 | 0 | 24 | 0 | 5 |
| Dianthidium (Dianthidium) cressonii | 50 | 4 | 0 | 46 | 5 |
| Dianthidium (Dianthidium) dubium | 0 | 0 | 0 | 0 | 1 |
| Dianthidium (Dianthidium) heterulkei | 1 | 1 | 0 | 0 | 0 |
| Dianthidium (Dianthidium) implicatum | 0 | 0 | 0 | 0 | 16 |
| Dianthidium (Dianthidium) parvum | 291 | 122 | 118 | 110 | 58 |
| Dianthidium (Dianthidium) platyurum | 24 | 6 | 34 | 1 | 11 |
| Dianthidium (Dianthidium) pudicum | 84 | 69 | 4 | 13 | 33 |
| Dianthidium (Dianthidium) ulkei | 248 | 127 | 42 | 100 | 54 |
| Dioxys pomonae | 19 | 3 | 0 | 16 | 6 |
| Heriades (Neotrypetes) cressoni | 251 | 5 | 72 | 210 | 106 |
| Heriades (Neotrypetes) micropthalma | 644 | 603 | 16 | 33 | 86 |
| Heriades (Neotrypetes) timberlakei | 575 | 203 | 152 | 296 | 157 |
| Heriades (Neotrypetes) variolosa | 1 | 0 | 0 | 1 | 0 |
| Hoplitis (Alcidamea) grinnelli | 2 | 1 | 0 | 1 | 26 |
| Hoplitis (Alcidamea) n. sp. 15 | 1 | 0 | 2 | 0 | 6 |
| Hoplitis (Alcidamea) producta | 14 | 4 | 14 | 3 | 1 |
| Hoplitis (Cyrtosmia) hypocrita | 10 | 0 | 8 | 6 | 2 |
| Hoplitis (Dasyosmia) paroselae | 1 | 1 | 0 | 0 | 12 |
| Hoplitis (Monumetha) albifrons | 22 | 1 | 40 | 1 | 6 |
| Hoplitis (Penteriades) incanescens | 76 | 4 | 0 | 72 | 6 |
| Hoplitis (Proteriades) n. sp. 1 (aff. shoshone) | 17 | 0 | 12 | 11 | 9 |
| Hoplitis (Proteriades) zuni | 198 | 89 | 104 | 57 | 31 |
| Lithurgus (Lithurgopsis) apicalis | 345 | 190 | 20 | 145 | 27 |
| Megachile (Argyropile) parallela | 38 | 24 | 12 | 8 | 50 |
| Megachile (Argyropile) rossi | 5 | 1 | 0 | 4 | 4 |
| Megachile (Argyropile) townsendiana | 0 | 0 | 0 | 0 | 3 |
| Megachile (Chelostomoides) prosopidis | 126 | 78 | 4 | 46 | 10 |
| Megachile (Chelostomoides) subexilis | 32 | 18 | 2 | 13 | 46 |
| Megachile (Eutricharaea) rotundata | 1 | 0 | 0 | 1 | 0 |
| Megachile (Litomegachile) coquilletti | 3 | 1 | 2 | 1 | 0 |
| Megachile (Litomegachile) lippiae | 71 | 38 | 38 | 14 | 13 |
| Megachile (Litomegachile) mendica | 14 | 5 | 10 | 4 | 1 |
| Megachile (Litomegachile) onobrychidis | 5 | 5 | 0 | 0 | 0 |
| Megachile (Litomegachile) sp. 2 (aff. brevis) | 89 | 72 | 4 | 15 | 4 |
| Megachile (Litomegachile) texana | 104 | 59 | 46 | 22 | 28 |
| Megachile (Megachile) montivaga | 9 | 4 | 2 | 4 | 1 |
| Megachile (Megachiloides) anograe | 1 | 1 | 0 | 0 | 13 |
| Megachile (Megachiloides) casadae | 28 | 10 | 2 | 17 | 2 |
| Megachile (Megachiloides) legalis | 55 | 21 | 0 | 34 | 6 |
| Megachile (Megachiloides) manifesta | 12 | 0 | 0 | 12 | 0 |
| Megachile (Megachiloides) micheneri | 1 | 0 | 0 | 1 | 0 |
| Megachile (Megachiloides) mucorosa | 2 | 2 | 0 | 0 | 0 |
| Megachile (Megachiloides) n. sp. 1 (aff. umatillensis) | 13 | 13 | 0 | 0 | 4 |
| Megachile (Megachiloides) nevadensis | 254 | 60 | 114 | 137 | 87 |
| Megachile (Megachiloides) sp. 1 | 1 | 0 | 0 | 1 | 0 |
| Megachile (Megachiloides) subanograe | 28 | 26 | 0 | 2 | 31 |
| Megachile (Megachiloides) sublaurita | 69 | 66 | 0 | 3 | 10 |
| Megachile (Megachiloides) subnigra | 28 | 7 | 6 | 18 | 7 |
| Megachile (Sayapis) fidelis | 4 | 1 | 2 | 2 | 5 |
| Megachile (Sayapis) inimica | 123 | 118 | 8 | 1 | 27 |
| Megachile (Sayapis) mellitarsis | 0 | 0 | 0 | 0 | 1 |
| Megachile (Sayapis) pugnata | 0 | 0 | 0 | 0 | 1 |
| Megachile (Xanthosarus) agustini | 26 | 1 | 0 | 25 | 2 |
| Megachile (Xanthosarus) cochisiana | 113 | 111 | 4 | 0 | 4 |
| Megachile (Xanthosarus) perihirta | 6 | 5 | 0 | 1 | 0 |
| Megachile (Xeromegachile) sp. 2 | 0 | 0 | 0 | 0 | 1 |
| Osmia (Acanthosmioides) alpestris | 4 | 0 | 0 | 4 | 0 |
| Osmia (Acanthosmioides) austromaritima | 7 | 0 | 0 | 7 | 0 |
| Osmia (Acanthosmioides) integra | 185 | 0 | 16 | 177 | 14 |
| Osmia (Acanthosmioides) longula | 2 | 0 | 0 | 2 | 0 |
| Osmia (Acanthosmioides) nigrifrons | 58 | 0 | 0 | 58 | 1 |
| Osmia (Acanthosmioides) unca | 30 | 1 | 14 | 22 | 0 |
| Osmia (Cephalosmia) californica | 114 | 0 | 8 | 110 | 1 |
| Osmia (Cephalosmia) grinnelli | 37 | 6 | 16 | 23 | 1 |
| Osmia (Cephalosmia) montana | 4 | 0 | 4 | 2 | 0 |
| Osmia (Cephalosmia) subaustralis | 1 | 0 | 0 | 1 | 0 |
| Osmia (Helicosmia) coloradensis | 165 | 19 | 10 | 141 | 2 |
| Osmia (Helicosmia) texana | 4 | 4 | 0 | 0 | 2 |
| Osmia (Melanosmia) albolateralis | 22 | 0 | 2 | 21 | 1 |
| Osmia (Melanosmia) austromaritima | 2 | 0 | 0 | 2 | 1 |
| Osmia (Melanosmia) brevis | 170 | 9 | 84 | 119 | 29 |
| Osmia (Melanosmia) bruneri | 188 | 0 | 10 | 183 | 5 |
| Osmia (Melanosmia) cerasi | 10 | 2 | 0 | 8 | 6 |
| Osmia (Melanosmia) clarescens | 3 | 1 | 0 | 2 | 1 |
| Osmia (Melanosmia) cobaltina | 150 | 0 | 16 | 142 | 2 |
| Osmia (Melanosmia) crassa | 12 | 11 | 0 | 1 | 8 |
| Osmia (Melanosmia) dakotensis | 32 | 4 | 0 | 28 | 3 |
| Osmia (Melanosmia) ednae | 8 | 0 | 8 | 4 | 8 |
| Osmia (Melanosmia) gaudiosa | 90 | 10 | 4 | 78 | 6 |
| Osmia (Melanosmia) iridis | 19 | 1 | 2 | 17 | 2 |
| Osmia (Melanosmia) kincaidii | 175 | 0 | 4 | 173 | 0 |
| Osmia (Melanosmia) liogastra | 6 | 0 | 0 | 6 | 0 |
| Osmia (Melanosmia) marginata | 2 | 0 | 0 | 2 | 4 |
| Osmia (Melanosmia) n. sp. 1 | 34 | 4 | 0 | 30 | 0 |
| Osmia (Melanosmia) n. sp. 2 (aff. enixa) | 13 | 6 | 0 | 7 | 6 |
| Osmia (Melanosmia) phenax | 14 | 2 | 0 | 12 | 31 |
| Osmia (Melanosmia) prunorum | 2 | 1 | 0 | 1 | 1 |
| Osmia (Melanosmia) pusilla | 2 | 1 | 0 | 1 | 0 |
| Osmia (Melanosmia) rawlinsi | 101 | 6 | 26 | 82 | 9 |
| Osmia (Melanosmia) sanrafaelae | 129 | 68 | 14 | 54 | 32 |
| Osmia (Melanosmia) sp. E1 | 0 | 0 | 0 | 0 | 1 |
| Osmia (Melanosmia) sp. E2 | 1 | 0 | 2 | 0 | 0 |
| Osmia (Melanosmia) sp. E3 | 0 | 0 | 0 | 0 | 1 |
| Osmia (Melanosmia) sp. E4 | 0 | 0 | 0 | 0 | 1 |
| Osmia (Melanosmia) sp. E5 | 2 | 0 | 4 | 0 | 1 |
| Osmia (Melanosmia) sp.2 (aff. cyanella) | 1 | 0 | 2 | 0 | 0 |
| Osmia (Melanosmia) trevoris | 191 | 0 | 0 | 191 | 2 |
| Osmia (Osmia) lignaria | 318 | 179 | 16 | 131 | 24 |
| Osmia (Osmia) ribifloris | 137 | 105 | 6 | 29 | 35 |
| Osmia (Trichinosmia) latisulcata | 15 | 7 | 4 | 6 | 2 |
| Protosmia (Chelostomopsis) rubifloris | 109 | 0 | 208 | 5 | 1 |
| Stelis (Dolichostelis) rudbeckiarum | 1 | 0 | 0 | 1 | 11 |
| Stelis (Stelis) anasazi | 4 | 1 | 0 | 3 | 1 |
| Stelis (Stelis) carnifex | 44 | 9 | 28 | 21 | 2 |
| Stelis (Stelis) imperialis | 1 | 0 | 0 | 1 | 0 |
| Stelis (Stelis) interrupta | 2 | 0 | 0 | 2 | 0 |
| Stelis (Stelis) lamelliterga | 9 | 1 | 14 | 1 | 0 |
| Stelis (Stelis) lateralis | 2 | 0 | 2 | 1 | 2 |
| Stelis (Stelis) montana | 3 | 0 | 0 | 3 | 0 |
| Stelis (Stelis) n. sp. 1 | 2 | 0 | 2 | 1 | 0 |
| Stelis (Stelis) n. sp. 2 | 0 | 0 | 0 | 0 | 1 |
| Stelis (Stelis) occidentalis | 0 | 0 | 0 | 0 | 3 |
| Stelis (Stelis) paiute | 1 | 0 | 0 | 1 | 0 |
| Stelis (Stelis) palmarum | 10 | 1 | 0 | 9 | 2 |
| Stelis (Stelis) pavonina | 0 | 0 | 0 | 0 | 1 |
| Stelis (Stelis) robertsoni | 1 | 0 | 0 | 1 | 1 |
| Stelis (Stelis) subemarginata | 1 | 0 | 2 | 0 | 0 |
| Trachusa (Heteranthidium) cordaticeps | 0 | 0 | 0 | 0 | 4 |
| Trachusa (Heteranthidium) zebrata | 1 | 0 | 2 | 0 | 0 |
| Hesperapis (Carinapis) carinata | 0 | 0 | 0 | 0 | 1 |
| Hesperapis (Carinapis) oliviae | 20 | 20 | 0 | 0 | 6 |
| Hesperapis (Disparapis) n. sp. 1 (aff. disparapis) | 0 | 0 | 0 | 0 | 5 |
| Hesperapis (Disparapis) sp. 1 (aff. cockerelli) | 0 | 0 | 0 | 0 | 33 |
| Hesperapis (Panurgomia) n. sp. 2 | 0 | 0 | 0 | 0 | 211 |
