# Supplementary material for: Reducing protected lands in a hotspot of bee biodiversity: bees of Grand Staircase-Escalante National Monument
Source: PeerJ. 2018 Dec 4;6:e6057. doi: 10.7717/peerj.6057 (PMC6284448; doi:10.7717/peerj.6057)
Supplement: Table S2 — Bee species no longer found in newly modified monument boundaries but formerly known from Grand Staircase Escalante National Monument. Distributions and other biological notes taken from Discoverlife.org (Ascher & Pickering, 2018). [file peerj-06-6057-s002.docx]

**Table S2.** Bee species no longer found in newly modified monument boundaries but formerly known from Grand Staircase Escalante National Monument. Distributions and other biological notes taken from Discoverlife.org (Ascher & Pickering 2018).

| **Family** | **Species** | **Number of specimens** | **Notes** |
| --- | --- | --- | --- |
| Andrenidae | Ancylandrena timberlakei | 1 | Known from the Mojave and Sonoran deserts, most in the genus are specialists |
| Andrenidae | Andrena (Callandrena) haynesi | 3 | Specialist on Heliantheae, known from the western Great Plains (east of the Rocky Mountains) |
| Andrenidae | Andrena (Callandrena) helianthi | 5 | Specialist on Asteraceae, prefers *Helianthus*, widespread across the US |
| Andrenidae | Andrena (Dactylandrena) porterae | 2 | Widespread in the west, specialist on *Ribes* |
| Andrenidae | Andrena (Micrandrena) melanochroa | 2 | Widespread generalist |
| Andrenidae | Andrena (Rhaphandrena) prima | 9 | Mainly known from southern NM and AZ and along the Colorado River up into the Mojave Desert, specialist on Brassicaceae, primarily *Physaria* (formally known as *Lesquerella*) |
| Andrenidae | Andrena (Scaphandrena) cruciferarum | 1 | specialist on Brassicaceae, known primarily from the interior coast range of California |
| Andrenidae | Andrena (Scaphandrena) sieverti | 6 | Rare, prior to GSENM only known from northern Utah and eastern WY |
| Andrenidae | Andrena (Trachandrena) salicifloris | 1 | generalist though shows a preference for *Salix*, found mainly in Northwestern states and California, often in montane areas, |
| Andrenidae | Calliopsis (Nomadopsis) puellae | 35 | specialist on *Malocothrix*, widespread |
| Andrenidae | Macrotera (Macroteropsis) arcuata | 1 | Mojave/Sonoran deserts, *Sphaeralcea* specialist |
| Andrenidae | Macrotera (Macroteropsis) latior | 1 | Mainly Chihuahuan/Mojave deserts, *Sphaeralcea* specialist |
| Andrenidae | Macrotera (Macroteropsis) n.sp. (aff. portalis) | 3 | Undescribed species |
| Andrenidae | Panurginus sp. 1 | 1 | Morpho- species |
| Andrenidae | Panurginus sp. 2 | 6 | Morpho- species |
| Andrenidae | Perdita sp. E10 | 5 | Morpho- species |
| Andrenidae | Perdita sp. E5 | 7 | Morpho- species |
| Andrenidae | Perdita sp. E8 | 1 | Morpho- species |
| Andrenidae | Perdita sp. E9 | 27 | Morpho- species |
| Andrenidae | Perdita (Cockerellia) lingualis | 3 | Widespread *Helianthus* specialist, Colorado Plateau and midwestern grasslands |
| Andrenidae | Perdita (Epimacrotera) crassula | 2 | Rare, only known from Sonoran and Chihuahuan deserts |
| Andrenidae | Perdita (Perdita) croceipes | 11 | Colorado Plateau endemic |
| Andrenidae | Perdita (Perdita) crotonis | 1 | Widespread, specialist on *Croton* |
| Andrenidae | Perdita (Perdita) euzonata | 6 | Rare, Colorado Plateau endemic |
| Andrenidae | Perdita (Perdita) glabrescens | 1 | Only known from the Mojave |
| Andrenidae | Perdita (Perdita) n. sp. 12 (aff. cuspidata) | 13 | Undescribed species |
| Andrenidae | Perdita (Perdita) n. sp. 3 (aff. tortifoliae) | 1 | Undescribed species |
| Andrenidae | Perdita (Perdita) sp. 2 (aff. xanthochroa) | 7 | Morpho- species |
| Andrenidae | Perdita (Perdita) sp. 4 (aff. punctifera) | 5 | Morpho- species |
| Andrenidae | Perdita (Perdita) sp. 8 (aff. koebelei) | 3 | Morpho- species |
| Andrenidae | Perdita (Pygoperdita) fallugiae | 11 | Known mainly from Mojave/Sonoran |
| Andrenidae | Perdita (Pygoperdita) sp. 1 (aff. mohavensis) | 7 | Morpho- species |
| Andrenidae | Pseudopanugus (Pterosarus) sp. E4 | 1 | Morpho- species |
| Apidae | Anthophora (Anthophoroides) pueblo | 2 | New species (described in 2016) Excavates nests in sandstone, Only known in Colorado Plateau and Northwestern Mojave desert, floral generalist |
| Apidae | Anthophora (Pyganthophora) edwardsii | 1 | Widespread in the west, generalist but has some preference for *Astragalus*, early spring bee (overwinters as adult) |
| Apidae | Anthophorula (Anthophorula) albata | 30 | Known mainly from Mojave/Sonoran |
| Apidae | Anthophorula (Anthophorula) crenulata | 38 | Known mainly from the Colorado Plateau |
| Apidae | Diadasia vallicola | 1 | Known mainly from Mojave/Sonoran, specialist on *Sphaeralcea* |
| Apidae | Epeolus sp. 3 | 1 | Morpho- species |
| Apidae | Hexepeolus rhodogyne | 1 | Only known from the Mojave/Sonoran Deserts, Parasite on Acnylandrena (suggests an established Ancylandrena population) |
| Apidae | Melecta (Melecta) alexanderi | 1 | Great Basin/Colorado Plateau Endemic |
| Apidae | Melecta (Melecta) bohartorum | 2 | Mainly Mojave Desert and Colorado Plateau |
| Apidae | Melissodes (Callimelissodes) coloradensis | 2 | Colorado Plateau and Upper Midwest, preferrs sunflowers |
| Apidae | Melissodes (Callimelissodes) compositus | 1 | across the west, mainly colorado plateau and SE Arizona |
| Apidae | Melissodes (Eumelissodes) brevipyga | 2 | across the west but not common? |
| Apidae | Melissodes (Eumelissodes) illatus | 1 | Only known from the North eastern US (should check this one) |
| Apidae | Neolarra (Neolarra) verbesinae | 7 | Known mainly from warm deserts and colorado plateau |
| Apidae | Nomada (Centrias) sp. E2 | 8 | Morpho- species |
| Apidae | Nomada (Nomada) sp. E13 | 1 | Morpho- species |
| Apidae | Nomada (Nomada) sp. E23 | 4 | Morpho- species |
| Apidae | Nomada (Nomada) sp. E25 | 1 | Morpho- species |
| Apidae | Svastra (Epimelissodes) helianthelli | 6 | Mainly hot deserts and colorado Plateau, sunflower specialist |
| Apidae | Triepeolus helianthi | 4 | widespread |
| Apidae | Triepeolus morpho sp. 51 | 1 | Morpho- species |
| Colletidae | Colletes fulgidus | 1 | widespread in the west, rare from mojave and sonoran deserts |
| Halictidae | Dufourea harveyi | 1 | oligolege on Potentilla, commonly found in alpine and boreal areas |
| Halictidae | Dufourea sp. 1 (aff. saundersi) | 2 | Morpho- species |
| Halictidae | Lasioglossum (Dialictus) sedi | 1 | western US, not in Hot deserts |
| Halictidae | Lasioglossum (Dialictus) semibrunneum | 10 | Mainly chihuahuan desert and great plains |
| Halictidae | Lasioglossum (Evylaeus) cooleyi | 2 | primatively eusocial, widespread in the west |
| Megachilidae | Anthidium (Anthidium) duomarginatum | 1 | Recently discovered (described in 2013) found only in the Colorado plateau and some surrounding mountains (Wasatch and Arizona mountians). |
| Megachilidae | Ashmeadiella (Arogochila) n. sp. 1 (aff. leachi) | 2 | Undescribed species |
| Megachilidae | Ashmeadiella (Ashmeadiella) sp. 1 (aff. rufipes) | 4 | Morpho- species |
| Megachilidae | Ashmeadiella (Ashmeadiella) sp. 2 (aff. titusi) | 11 | Morpho- species |
| Megachilidae | Ashmeadiella (Cubitognatha) xenomastax | 48 | Mainly known from mojave and sonoran deserts |
| Megachilidae | Coelioxys (Cyrtocoelioxys) gilensis | 5 | widespread, parasite of leaf cutter bees |
| Megachilidae | Coelioxys (Synocoelioxys) apacheorum | 1 | widespread, parasite of leaf cutter bees |
| Megachilidae | Dianthidium (Dianthidium) dubium | 1 | mainly known from the mountains of california, rare east of California |
| Megachilidae | Dianthidium (Dianthidium) implicatum | 16 | widespread in west |
| Megachilidae | Megachile (Argyropile) townsendiana | 3 | Hot deserts primarily |
| Megachilidae | Megachile (Sayapis) mellitarsis | 1 | Primarily found in Colorado Plateau and Great Basin |
| Megachilidae | Megachile (Sayapis) pugnata | 1 | widespread across the US |
| Megachilidae | Megachile (Xeromegachile) sp. 2 | 1 | Morpho- species |
| Megachilidae | Osmia (Melanosmia) sp. E1 | 1 | Morpho- species |
| Megachilidae | Osmia (Melanosmia) sp. E3 | 1 | Morpho- species |
| Megachilidae | Osmia (Melanosmia) sp. E4 | 1 | Morpho- species |
| Megachilidae | Stelis (Stelis) n. sp. 2 | 1 | Undescribed species |
| Megachilidae | Stelis (Stelis) occidentalis | 3 | widesperad in the west, primarily in mountains and Mojave Desert |
| Megachilidae | Stelis (Stelis) pavonina | 1 | Montain bee known from Rocky Mountains and Sierra Nevada Mountains |
| Megachilidae | Trachusa (Heteranthidium) cordaticeps | 4 | Mainly known from Chihuahuan and Sonoran deserts. |
| Melittidae | Hesperapis (Carinapis) carinata | 1 | widespread in west though uncommon |
| Melittidae | Hesperapis (Disparapis) n. sp. 1 (aff. disparapis) | 5 | Undescribed species |
| Melittidae | Hesperapis (Disparapis) sp. 1 (aff. cockerelli) | 33 | Morpho- species |
| Melittidae | Hesperapis (Panurgomia) n. sp. 2 | 211 | Undescribed species |
